# Comment on the manuscript entitled "Clinical presentation vs endoscopy for an early diagnosis of eosinophilic esophagitis: a case report" by Di Stefano et al.

**DOI:** 10.17179/excli2022-5695

**Published:** 2023-01-06

**Authors:** Alejandro José Quiroz Alfaro, Juliana Greiffenstein Florez, Catalina Andrea Dussan Tovar

**Affiliations:** 1 Universidad Colegio Mayor de Nuestra Señora del Rosario, Bogotá, Colombia; 2 Universidad Pontificia Bolivariana, Medellín, Colombia; 3 Epidemiology, Universidad de La Sabana, Chía, Colombia

## ⁯⁯⁯

We have read the case report entitled “Clinical presentation vs endoscopy for an early diagnosis of eosinophilic esophagitis: a case report” by Di Stefano et al. (2022[1]). We would like to congratulate the authors for their excellent manuscript and make some contributions.

In the case report (Di Stefano et al., 2022[1]), the authors claim that the sampling of mucosa from the esophagus is not a routinely performed procedure during endoscopy and that a macroscopically "normal" appearing mucosa has a low negative predictive value. Thus, to diagnose esophageal conditions characterized by histological lesions and macroscopically normal mucosa, such as eosinophilic esophagitis (EoE), esophageal biopsy specimens should be collected based more on clinical reasons rather than endoscopic appearance, especially in adults with dysphagia. We agree with the authors; furthermore, a retrospective study by Jung et al. (2010[2]) analyzing 1,609 biopsy specimens found that 0.4 % of the total biopsied cases (Korean patients with atypical endoscopic findings and symptoms mimicking gastroesophageal reflux) had EoE. Another study by Miller et al. (2011[3]) claimed that routine esophageal mucosal sampling in patients with gastroesophageal reflux refractory to proton pump inhibitors may even be cost-effective when the prevalence of EoE exceeds 8 %. Therefore, we consider that mucosal sampling of the esophagus should be determined on clinical reasons rather than endoscopic findings.

On the other hand, a systematic review by Shaheen et al. (2018[4]), which compiled the epidemiological and natural history data on EoE from 47 manuscripts, showed that, as a symptom, food impaction occurred between 6.7 % to 21.7 % in children overall and between 16.9 % to 65.7 % in adults. Food impactions were more common in men (43 %) than in women (28 %), with variations in distribution according to race, being more common in Caucasians (35 %) and African Americans (13 %) than in other races (13 %). Although food impaction is a common symptom in EoE patients (up to 65.7%), it should be noted that no study found it in 100 % of patients; therefore, it should not be considered a pathognomonic symptom of EoE.

## Declaration

### Conflict of interest

The authors declare no conflicts of interest.

### Financial disclosures

None. 
